# Genetic Variation of the SusC/SusD Homologs from a Polysaccharide Utilization Locus Underlies Divergent Fructan Specificities and Functional Adaptation in *Bacteroides thetaiotaomicron* Strains

**DOI:** 10.1128/mSphereDirect.00185-18

**Published:** 2018-05-23

**Authors:** Payal Joglekar, Erica D. Sonnenburg, Steven K. Higginbottom, Kristen A. Earle, Carl Morland, Sarah Shapiro-Ward, David N. Bolam, Justin L. Sonnenburg

**Affiliations:** a Department of Microbiology and Immunology, Stanford University School of Medicine, Stanford, California, USA; b Institute for Cell and Molecular Biosciences, Newcastle University, Medical School, Newcastle upon Tyne, United Kingdom; University of Iowa; Agriculture and Agri-Food Canada; University of Michigan Medical School

**Keywords:** *Bacteroides*, microbiome, polysaccharide utilization locus

## Abstract

Genomic differences between gut-resident bacterial strains likely underlie significant interindividual variation in microbiome function. Traditional methods of determining community composition, such as 16S rRNA gene amplicon sequencing, fail to capture this functional diversity. Metagenomic approaches are a significant step forward in identifying strain-level sequence variants; however, given the current paucity of biochemical information, they too are limited to mainly low-resolution and incomplete functional predictions. Using genomic, biochemical, and molecular approaches, we identified differences in the fructan utilization profiles of two closely related Bacteroides thetaiotaomicron strains. B. thetaiotaomicron 8736 (*Bt-8736*) contains a fructan polysaccharide utilization locus (PUL) with a divergent *susC*/*susD* homolog gene pair that enables it to utilize inulin, differentiating this strain from other characterized *Bt* strains. Transfer of the distinct pair of *susC*/*susD* genes from *Bt-8736* into the noninulin using type strain B. thetaiotaomicron* VPI-5482* resulted in inulin use by the recipient strain, *Bt*(*8736-2*). The presence of the divergent *susC*/*susD* gene pair alone enabled the hybrid *Bt*(*8736-2*) strain to outcompete the wild-type strain *in vivo* in mice fed an inulin diet. Further, we discovered that the *susC*/*susD* homolog gene pair facilitated import of inulin into the periplasm without surface predigestion by an endo-acting enzyme, possibly due to the short average chain length of inulin compared to many other polysaccharides. Our data builds upon recent reports of dietary polysaccharide utilization mechanisms found in members of the *Bacteroides* genus and demonstrates how the acquisition of two genes can alter the functionality and success of a strain within the gut.

**IMPORTANCE** Dietary polysaccharides play a dominant role in shaping the composition and functionality of our gut microbiota. Dietary interventions using these microbiota-accessible carbohydrates (MACs) serve as a promising tool for manipulating the gut microbial community. However, our current gap in knowledge regarding microbial metabolic pathways that are involved in the degradation of these MACs has made the design of rational interventions difficult. The issue is further complicated by the diversity of pathways observed for the utilization of similar MACs, even in closely related microbial strains. Our current work focuses on divergent fructan utilization pathways in two closely related B. thetaiotaomicron strains and provides an integrated approach to characterize the molecular basis for strain-level functional differences.

## Abstract (precis)

Dietary polysaccharides play a dominant role in shaping the composition and functionality of our gut microbiota. Dietary interventions using these microbiota-accessible carbohydrates (MACs) serve as a promising tool for manipulating the gut microbial community. However, our current gap in knowledge regarding microbial metabolic pathways that are involved in the degradation of these MACs has made the design of rational interventions difficult. The issue is further complicated by the diversity of pathways observed for the utilization of similar MACs, even in closely related microbial strains. Our current work focuses on divergent fructan utilization pathways in two closely related B. thetaiotaomicron strains and provides an integrated approach to characterize the molecular basis for strain-level functional differences.

## INTRODUCTION

A key step toward assessing gut microbiota structure and dynamics is to define the diversity that encompasses this complex community. A gold standard for gut microbiota profiling is the 16S rRNA gene amplicon sequencing method, followed by either taxonomical classification or *de novo* clustering into operational taxonomic units (OTUs) at 97% identity. This identity cutoff value is based on the premise that phylogenetic relatedness can serve as a proxy for whole genomes and assumes similar functional profiles above this predetermined cutoff value (1). More-recent studies using novel single-nucleotide-resolution approaches for cross-sample comparisons of 16S rRNA gene amplicons such as oligotyping (2) and cluster-free sub-OTU analysis (3) showed that even sequences that share >99.0% identity at the 16S rRNA locus can occupy distinct ecological niches, thus representing unique strains or ecotypes with distinct functionalities. In recent years, an explosion in whole-genome sequencing of representative bacterial strains from diverse ecosystems has revealed substantial intraspecies gene content variation (4). Although the sizes of the “accessory genomes” differ greatly between species (5), it is safe to assume that the functions encoded by a species that is present in the gut microbiome would be highly strain dependent. Shotgun metagenomics allows higher resolution than the traditional marker gene approaches, thus offering a greater potential for identification of strain-level genomic variation (6). However, this approach too is limited by our current lack of biochemical data linking genetic variants to actual functional differences.

In our current study, we identified the genetic basis for the observed differences in fructan utilization capabilities between two B. thetaiotaomicron strains. The study highlights the need to combine omics-based techniques with traditional culture-based biochemical, structural, and molecular tools to identify and characterize the functional diversity encoded by our gut microbes. B. thetaiotaomicron VPI-5482, a type strain for the species, carries a polysaccharide utilization locus (PUL) responsible for fructan utilization (7). This previously characterized PUL encodes (i) family 32 glycoside hydrolases (GH32s) for glycosidic linkage degradation (8), (ii) outer membrane proteins (SusC/SusD homologs and a surface glycan binding protein [SGBP]) for glycan recognition and import (9), and (iii) a hybrid two-component system (HTCS) sensor regulator for activating expression of the PUL in response to dietary fructose (10). The PUL is specific for degradation and uptake of β2-6-linked fructans (levan) (7). In this study, we identified a closely related B. thetaiotaomicron strain (*Bt-8736*) that utilizes β2-1-linked fructans (inulin) instead of levan and that outcompeted B. thetaiotaomicron* VPI-5482 in vivo*, in the presence of dietary inulin. Whole-genome sequencing, followed by sequence analysis, was used to identify a putative fructan PUL in *Bt-8736*. The two fructan PULs differ in the genes that are known to encode fructan specificity. Using genetic and biochemical approaches, coupled with *in vivo* testing, we were able to demonstrate the inulin specificity of the *Bt-8736* gene products. Further, the current work reveals that the mechanism of inulin utilization is distinct from levan use, as it occurs in the absence of any cell surface endo-acting enzymes. Instead, *Bt-8736* can directly import inulin into its periplasm for intracellular degradation, possibly due to the short average chain length of inulin compared to levan and other polysaccharides. Our current work demonstrates that related genetic loci can encode diversified biochemical pathways in closely related gut commensal strains. The pathways incorporate enzymes and carbohydrate binding and import proteins with distinct substrate specificities, which could not have been predicted previously based on sequence data alone (11).

## RESULTS

### *Bt-8736* encodes a fructan utilization PUL that confers inulin use.

Many members of the *Bacteroides* genus carry a polysaccharide utilization locus (PUL) that encodes the apparatus necessary for uptake and degradation of fructose-based dietary polysaccharides, collectively known as fructans (7). In a previous study, using B. thetaiotaomicron* VPI-5482* as a prototypic member of this genus, we defined the genetic and biochemical basis of fructan PUL specificity. That work demonstrated that the fructan PUL is variably conserved among the *Bacteroides* species, resulting in functional differences in the fructan utilization profiles across the genus. The ecological relevance of these functional differences was demonstrated using gnotobiotic mice harboring defined, two-member gut communities, where dietary fructan dictated the community composition, depending on the fructan degradation capacities of individual members of the microbiota (7).

**FIG 1:**
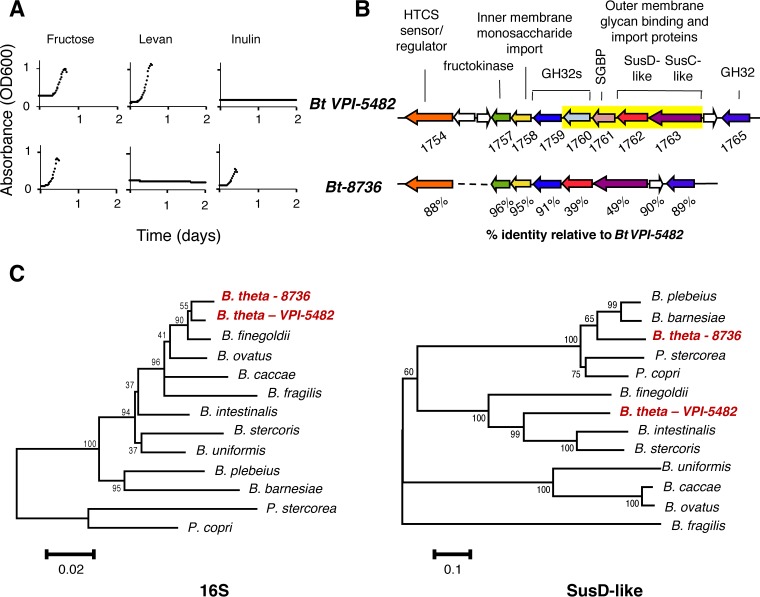
*Bt-8736* possesses a fructan PUL that enables inulin use. (A) Growth curve analysis of B. thetaiotaomicron* VPI-5482* (top) and *Bt-8736* (bottom) in minimal medium containing the indicated carbon source at 0.5% (wt/vol). (B) Comparative analysis of the putative fructan PUL from *Bt-8736* with the well-characterized levan PUL from B. thetaiotaomicron* VPI-5482* (*Bt VPI-5482*). Matching gene colors indicate homologs between the two genomes. The yellow box represents genes in the fructan PUL of B. thetaiotaomicron* VPI-5482* that either lack orthologs in *Bt-8736* or show a low degree of homology with the orthologous genes that are present; amino acid identity is indicated for orthologous genes. (C) Phylogenetic relationship of the 16S rRNA gene sequence (left) and the fructan PUL SusD homolog amino acid sequence (right) for indicated species and strains. Sequences from the two B. thetaiotaomicron strains are highlighted in red. Branch numbers represent percentages of bootstrap values in 1,000 sampling replicates, and the scale depicts branch lengths. *B. theta*, Bacteroides thetaiotaomicron; *B. finegoldii*, Bacteroides finegoldii; *B. ovatus*, Bacteroides ovatus; *B. caccae*, Bacteroides caccae; *B. fragilis*, Bacteroides fragilis; *B. intestinalis*, Bacteroides intestinalis; *B. stercoris*, Bacteroides stercoris; *B. uniformis*, Bacteroides uniformis; *B. plebeius*, Bacteroides plebeius; *B. barnesiae*, Bacteroides barnesiae; *P. stercorea*, Prevotella stercorea; *P. copri*, Prevotella copri.

Here we have assessed whether strains within a given *Bacteroides* species display similar fructan utilization profiles. We carried out an *in vitro* growth analysis of B. thetaiotaomicron strains present in our culture collection, in the presence of *Bacteroides* minimal medium (MM) containing either levan (0.5% [wt/vol]) or inulin (0.5% [wt/vol]) as the sole carbon source. These two polysaccharides were selected because they represent two distinct glycosidic linkages (β2-6 in levan and β2-1 in inulin) that are present in the fructan homopolymers and that are available to the gut microbiota. We identified one strain, *Bt-8736*, which, unlike all other strains tested (including the type strain, B. thetaiotaomicron* VPI-5482*), grew in inulin but not levan (Fig. 1A). To identify the genetic basis that defines this β2-1 specificity, the *Bt-8736* genome was sequenced, and a single fructan PUL orthologous to the one present in the B. thetaiotaomicron* VPI-5482* type strain was identified (Fig. 1B). Induction of this putative fructan PUL in the presence of fructose was confirmed by quantitative reverse transcription-PCR (qRT-PCR) (see Fig. S1 in the supplemental material).

10.1128/mSphereDirect.00185-18.1 FIG S1  *Bt-8736* regulates its fructan PUL in response to fructose. The figure shows results of gene expression analysis of the putative fructan PUL genes in *Bt*-8736 in MM fructose relative to expression in MM glucose. Download FIG S1, TIF file, 0.5 MB. Copyright © 2018 Joglekar et al. 2018 Joglekar et al. This content is distributed under the terms of the Creative Commons Attribution 4.0 International license.

A closer look at the PUL encoded by the *Bt-8736* genome revealed that it contained an HTCS, a putative fructokinase and inner membrane monosaccharide importer, and GH32 genes that are orthologs of *BT1759* and *BT1765*, supporting the idea of a fructan-associated role for this operon. Notably absent, relative to the B. thetaiotaomicron* VPI-5482* strain, was an ortholog of BT1760, a GH32 cell surface endo-levanase, and an ortholog of BT1761, a surface glycan binding protein, both of which exhibit specificity for the β2-6 linkage found in levan (7). GH32 enzymes are known to be active only on fructans and to display either endo-activity or exo-activity against either β2-1-linked or β2-6-linked fructans (12). Our previous work had shown that the presence of the cell surface levanase of B. thetaiotaomicron* VPI-5482* was critical for the ability of this strain to use the levan (7). On the basis of this information, our current data indicate that lack of a *BT1760* ortholog contributes to the inability of *Bt-8736* to utilize levan. Along with GH32s, the *susC*/*susD* homolog genes that encode the outer membrane TonB-dependent transporter (TBDT; SusC-like) and associated carbohydrate binding surface lipoprotein (SusD-like) (9, 13) also determine the specificity of the fructans that can be utilized. Accordingly, the SusC/D homologs from the fructan PULs of the three sequenced B. thetaiotaomicron strains (*Bt-3731*, *Bt-7330*, *and* B. thetaiotaomicron* VPI-5482*) (each with the confirmed ability to utilize levan) exhibit a high level of conservation (all with >97% amino acid identity in pairwise comparisons) (data not shown). On the other hand, the SusC/D homolog pair from the inulin-utilizing *Bt-8736* strain showed a striking lack of conservation compared to B. thetaiotaomicron* VPI-5482* (49% and 39% amino acid identity, respectively). Phylogenetic analysis of the divergent SusC/D homologs present in the two PULs revealed that, on the basis of the traditional 16S rRNA gene analysis, their genes indeed do not track with the evolutionary history of the B. thetaiotaomicron strains (Fig. 1C; see also Fig. S2A). Furthermore, isothermal titration calorimetry (ITC) performed with a recombinant form of *Bt-8736* SusD confirmed that the protein bound inulin but not levan (Fig. S2B).

10.1128/mSphereDirect.00185-18.2 FIG S2  Lack of orthology between the fructan PUL SusC homologs in two B. thetaiotaomicron strains and fructan specificity of *Bt-8736* SusD homolog. (A) Phylogenetic relationships of the fructan PUL SusC homolog amino acid sequences for the indicated species and strains. (B) ITC showing that the SusD homolog from the *Bt-8736* fructan PUL binds inulin but not levan. Download FIG S2, TIF file, 1.2 MB. Copyright © 2018 Joglekar et al. 2018 Joglekar et al. This content is distributed under the terms of the Creative Commons Attribution 4.0 International license.

**FIG 2:**
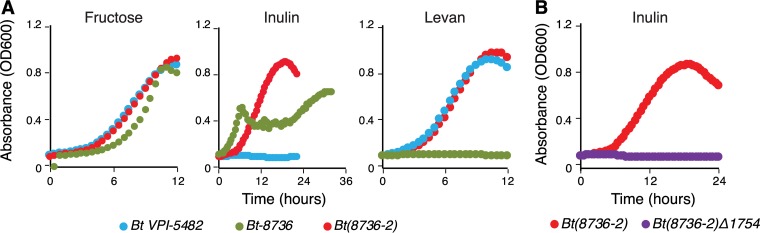
The *susC*/*susD* homolog pair from the fructan PUL of *Bt-8736* enabled usage of inulin in the B. thetaiotaomicron type strain. (A) Growth curve analysis of B. thetaiotaomicron strains in MM containing the indicated carbon source at 0.5% (wt/vol). (B) Growth curve analysis of *Bt*(*8736-2*)*Δ1754* in MM containing inulin (0.5% [wt/vol]).

To test whether the divergent SusC/D homologs dictate β2-1 linkage specificity in *Bt-8736*, we transferred the *susC*/*susD* homolog genes, along with the upstream intergenic region carrying a putative promoter sequence, into the levan-utilizing strain, B. thetaiotaomicron* VPI-5482* (Fig. S3A). Interestingly, despite the observed low homology between the SusC/D homologs of the two PULs, the putative promoter sequence from *Bt-8736* displayed 91% identity with the sequence of the corresponding promoter found upstream of the *BT1763* gene in B. thetaiotaomicron* VPI-5482*, indicative of promoter conservation at the species level. The presence of the SusC/D homologs from *Bt-8736* enabled inulin utilization by the resultant transgenic strain, *Bt*(*8736-2*), without affecting its ability to utilize levan, as tested by its growth in MM containing either inulin (0.5% [wt/vol]) or levan (0.5% [wt/vol]) (Fig. 2A). Expression of the transferred *Bt-8736* genes, along with the native fructan PUL genes of B. thetaiotaomicron* VPI-5482* (fructokinase, *BT1757*; *susC* homolog, *BT1763*) was confirmed in MM-fructose (0.5% [wt/vol]), with fructose being a known inducer of the fructan PUL (7) (Fig. S3B). Notably, the growth kinetics of *Bt-8736* and *Bt*(*8736-2*) in MM-inulin are reproducibly distinct; however, the basis for this difference remains to be determined. Further, transfer of the *susC*/*susD* homologs in a previously generated B. thetaiotaomicron* VPI-5482* mutant (7), lacking the native hybrid two-component sensor regulator (HTCS) gene (*BT1754*), prevented growth on inulin (and other fructans; data not shown) (Fig. 2B).

10.1128/mSphereDirect.00185-18.3 FIG S3  Fructose-induced expression of *susC*/*susD* homologs from *Bt-8736* fructan PUL upon transfer into the type strain. (A) Fructan utilization genes in the transgenic type strain, *Bt*(*8736-2*), after the transfer of *susC*/*susD* homologs from the *Bt-8736* fructan PUL into B. thetaiotaomicron* VPI-5482*. The expanded fructan machinery consists of the native PUL of B. thetaiotaomicron* VPI-5482* and the transferred homolog pair along with its own promoter. (B) Gene expression analysis of the expanded fructan PUL genes in *Bt*(*8736-2*) in MM fructose relative to their expression in MM glucose. Download FIG S3, TIF file, 1.3 MB. Copyright © 2018 Joglekar et al. 2018 Joglekar et al. This content is distributed under the terms of the Creative Commons Attribution 4.0 International license.

Together, these data confirm that the fructan PUL from *Bt-8736* lacks the genes required for effective levan use. Instead, the divergent *susC*/*susD* homolog genes confer inulin specificity to *Bt-8736*, and to *Bt*(*8736-2*), when they were transferred along with their native promoter region.

### **The***** susC***/***susD***** homolog genes from the fructan PUL of** ***Bt-8736***** improve accessibility of periplasmic exo-GH32s to inulin.**

**FIG 3:**
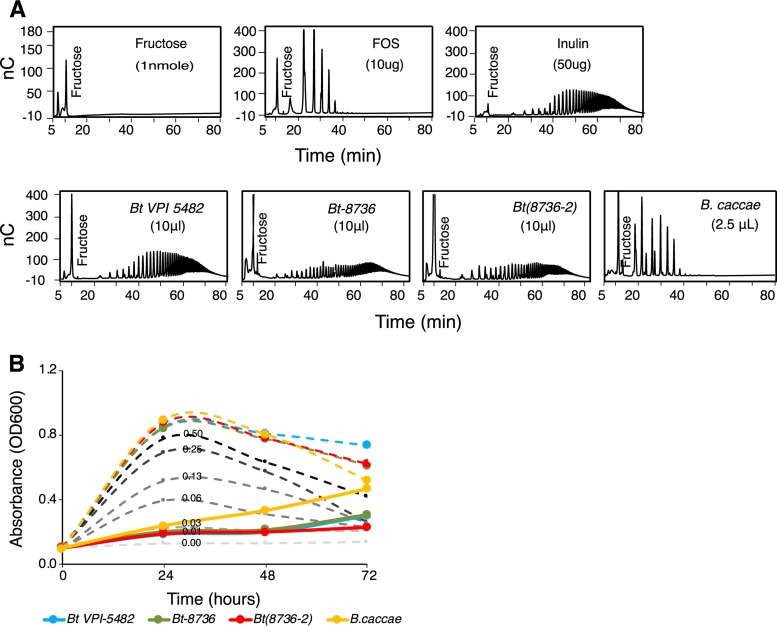
SusC/D homologs from the fructan PUL of *Bt-8736* facilitate growth on inulin without extracellular degradation. (A) HPAEC-PAD analysis of the products of surface inulin digestion by whole cells shown for each bacterium. Controls (fructose, fructo-oligosaccharides [FOS], and inulin) are shown. nC, nanoCoulomb. (B) Growth curve analysis of B. thetaiotaomicron* VPI-5482* in reconstituted spent MM-inulin (0.5% [wt/vol]), derived from mid-log-phase cultures of the strains indicated in the figure. The spent medium was either supplemented (dashed lines) with fructose (0.5% [wt/vol]) or left unsupplemented (solid lines). Gray dashed lines represent control growth curves of B. thetaiotaomicron* VPI-5482* in MM containing fructose at the indicated concentrations (%[wt/vol]).

To provide insight into the mechanism of inulin use by *Bt-8736* and the transgenic type strain *Bt*(*8736-2*), the cells were grown in MM-fructose to the mid-log phase in order to induce the expression of their fructan PULs. Washed cells were then incubated in phosphate-buffered saline (PBS) (1×) containing inulin (0.5% [wt/vol]) under growth-arresting aerobic conditions and were monitored for cell surface inulin degradation activity via the appearance of mono-, di-, tri-, and oligosaccharides using high-performance anion-exchange chromatography with pulsed amperometric detection (HPAEC-PAD). The HPAEC data showed that incubation of the whole cells with inulin resulted in the generation of low levels of free fructose in all the three B. thetaiotaomicron strains tested (Fig. 3A; see also Fig. S4). Notably, inulin degradation by these strains was not accompanied by the short-chain fructo-oligosaccharides generated by species that express endo-inulinases on the cell surface, such as Bacteroides caccae (Fig. 3A; see also Fig. S4).

10.1128/mSphereDirect.00185-18.4 FIG S4  Cell surface activity of B. thetaiotaomicron strains against inulin. The figure shows results of TLC analysis of the products of cell surface inulin digestion of whole cells grown in MM-fructose. F, fructose; S, sucrose; MT, maltotriose; FOS, fructo-oligosaccharides; In, inulin. Download FIG S4, TIF file, 2.7 MB. Copyright © 2018 Joglekar et al. 2018 Joglekar et al. This content is distributed under the terms of the Creative Commons Attribution 4.0 International license.

We next tested whether the generation of small amounts of free fructose contributes to the growth of the *Bt-8736* and *Bt*(*8736-2*) strains on MM-inulin. B. thetaiotaomicron* VPI-5482*, which is able to utilize fructose as a sole carbon source, was cultured in reconstituted, filter-sterilized spent media, derived from mid-log cultures of *Bt-8736* and *Bt*(*8736-2*) in MM-inulin (0.5% [wt/vol]). Spent media from B. thetaiotaomicron* VPI-5482*, an inulin nonutilizer, and B. caccae, which releases short-chain fructo-oligosaccharides, served as controls. Our data showed that the spent media derived from the B. thetaiotaomicron strains were incapable of supporting the growth of B. thetaiotaomicron* VPI-5482* (Fig. 3B). However, addition of fructose at a 0.5% (wt/vol) final concentration to the reconstituted spent media allowed the growth of B. thetaiotaomicron* VPI-5482*, supporting that the level of extracellular fructose generated by the B. thetaiotaomicron strains during inulin degradation was not sufficient to support growth.

**FIG 4:**
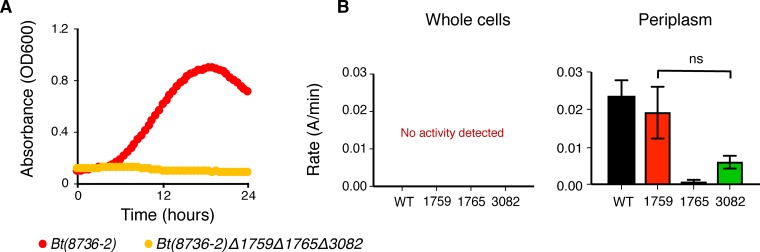
*Bt*(8736-2) utilizes periplasmic exo-fructosidases from B. thetaiotaomicron* VPI-5482* to break down inulin. (A) Growth curve analysis of *Bt*(*8736-2*)*Δ1759Δ1765Δ3082* in MM containing inulin (0.5% [wt/vol]). (B) Hydrolysis of inulin (0.5% [wt/vol] and 1.0% [wt/vol]—data combined) analyzed in whole-cell fractions (i.e., cell surface activity only) and periplasmic fractions by using B. thetaiotaomicron* VPI-5482* GH32 double-knockout mutants. The remaining exo-acting GH32 is indicated for each strain assayed. WT, wild type.

We then went on to test if the transgenic strain, *Bt*(*8736-2*), was reliant upon the native GH32s encoded by the B. thetaiotaomicron* VPI-5482* genome for inulin degradation. We previously have shown that B. thetaiotaomicron* VPI-5482* encodes three exo-acting fructosidases that release fructose from both β2-1-linked and β2-6-linked fructans (7). We tested the requirement of these endogenous GHs by transferring the *susC*/*susD* homologs from *Bt-8736* into a B. thetaiotaomicron* VPI-5482* mutant strain that lacked all three native GH32 genes (*BT1759*, *BT1765*, and *BT3082*). The resulting mutant, *Bt*(*8736-2*)*Δ1759Δ1765Δ3082*, was unable to grow on MM-inulin (Fig. 4A). Therefore, at least one of the GH32s from B. thetaiotaomicron* VPI-5482* is required for inulin degradation, and the SusC/D homologs from *Bt-8736* allow access of these enzymes to extracellular inulin. To test this idea, we generated GH32 double-knockout strains of B. thetaiotaomicron* VPI-5482* such that each mutant expresses only one exo-acting GH32. Subcellular fractionation of these mutants, followed by enzymatic assays performed with inulin (0.5 [wt/vol] and 1% [wt/vol]; data combined), confirmed that inulin was being degraded in the periplasm and that BT1759 and BT3082 together were mainly responsible for the inulin degradation in these cells (Fig. 4B). Notably, *Bt-8736* encodes close homologs of all three B. thetaiotaomicron* VPI-5482* fructosidases but lacks any putative endo-acting inulinases. Collectively, these data support a model of inulin use in which the SusC/D homologs from *Bt-8736* enable transport of inulin across the outer membrane, thus making the fructan accessible to the exo-acting GH32 enzymes localized in its periplasm.

### **The fructan PUL** ***susC***/***D***** genes from** ***Bt-8736***** are sufficient to drive a diet-dependent functional advantage** ***in vivo.***

**FIG 5:**
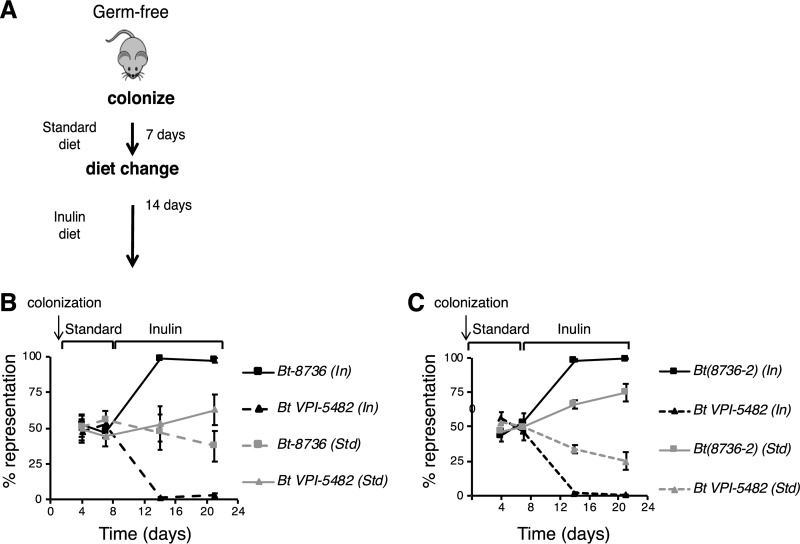
The divergent fructan PUL SusC/D pair alone conferred a diet-dependent functional advantage to *Bt*(*8736-2*) *in vivo*. (A) Experimental design for *in vivo* experiments. (B) Average relative fecal proportions (percentages of total bacteria) of *Bt-8736* and B. thetaiotaomicron* VPI-5482* on days 4, 7, 14, and 21 in samples from cocolonized mice fed a standard diet (Std) for the first 7 days followed by an inulin diet (In) for the next 14 days or fed a standard diet throughout the 21-day period. (C) Average relative fecal proportions (percentages of total bacteria) of B. thetaiotaomicron* VPI-5482* and *Bt*(8736-2) on days 4, 7, 14, and 21 after cocolonization in germfree mice.

Differences in the ability to consume host dietary carbohydrates can dictate competitiveness within members of the gut microbiota, favoring those endowed with the capacity to utilize existing dietary microbiota-accessible carbohydrates (MACs) (7). We therefore set out to determine whether the difference in inulin utilization capacity between *Bt-8736* and B. thetaiotaomicron* VPI-5482* resulted in a difference in bacterial fitness *in vivo*. Germfree mice (*n* = 12) were cocolonized with equivalent quantities of *Bt-8736* and B. thetaiotaomicron* VPI-5482* and were fed a standard diet containing diverse MACs for 7 days. Four mice were then switched to a diet in which inulin was the sole carbohydrate source for 14 days (Fig. 5A). Mice were individually housed throughout the experiment to prevent cross inoculation, and the relative abundances of the strains were assessed using strain-specific primers by quantitative PCR. Seven days after cocolonization, *Bt-8736* and B. thetaiotaomicron* VPI-5482* were present at equivalent densities (47.5 ± 3.5% and 52.5 ± 3.5%, respectively). However, 1 week after switching to the inulin diet, *Bt-8736* accounted for 98.6 ± 0.1% of the total population. This increase in abundance was maintained through day 21 (*Bt-8736* at 97.2 ± 1.7% and B. thetaiotaomicron* VPI-5482* at 2.8 ± 1.7% [*P* = 4.9 × 10^−5^, day 21 versus day 7; Student’s *t* test]) (Fig. 5B). The relative abundances of *Bt-8736* and B. thetaiotaomicron* VPI-5482* in cocolonized mice fed a standard diet were not statistically different even after 21 days (*P* = 0.7, day 21 versus day 7; Student’s *t* test, Fig. 5B). The *susC* and *susD* homologs of the fructan PUL from *Bt-8736* were upregulated on day 21 (14 days after the introduction of dietary inulin) relative to day 7 (9.7 ± 0.17-fold and 22.8 ± 0.19-fold, respectively), indicating that *Bt-8736* was actively sensing inulin and upregulating the inulin utilization machinery in the presence of dietary inulin. These data are consistent with a model in which the presence of a specific MAC may allow a bacterial strain to gain an advantage over a closely related strain within the gut due to small differences in gene content that confer the ability to utilize that MAC (7).

On the basis of our *in vitro* data, we then set out to test whether the presence of the β2-1-linkage-specific SusC/D homologs alone was sufficient to confer an *in vivo* advantage to a strain in the presence of dietary inulin. Germfree mice (*n* = 8) were cocolonized with wild-type B. thetaiotaomicron* VPI-5482* and the modified mutant strain harboring the *Bt-8736 susC*/*D* [*Bt*(*8736-2*)]. The experimental setup was similar to that outlined in Fig. 5A. B. thetaiotaomicron* VPI-5482* and *Bt*(8736-2) started out at similar densities. Upon a shift to the inulin diet, *Bt*(*8736-2*) reached relative densities of 98.1 ± 1.0% on day 14 and 99.3 ± 0.4% on day 21 (*P* = 4.6 × 10^−4^, day 21 versus day 7; Student’s *t* test) (Fig. 5C). Notably, *Bt*(*8736-2*) also exhibited a significant, though less striking, competitive advantage over the wild-type B. thetaiotaomicron* VPI-5482* strain with the MAC-rich standard diet over 21 days [49.6 ± 1.5% to 74.8 ± 6.0% relative abundance of *Bt*(*8736-2*) on day 7 to day 21; *P* = 7.6 × 10^−3^, Student’s *t* test], possibly reflecting the presence of β2-1-linked fructans in the standard mouse diet.

Our *in vivo* data confirmed that two wild-type strains of B. thetaiotaomicron, *Bt-8736* and B. thetaiotaomicron* VPI-5482*, have divergent fructan degradation capabilities attributable to the different specificities in the SusC/D homologs encoded by their respective fructan utilization PULs and that this functional difference provides a competitive advantage to *Bt-8736 in vivo* in the presence of dietary inulin.

## DISCUSSION

Our current work identified genetic variation in a polysaccharide utilization locus in two B. thetaiotaomicron strains, B. thetaiotaomicron* VPI-5482* and *Bt-8736*, which results in differences in the type of fructans that are utilized by these two closely related organisms. These functional differences dictated interstrain competition in a simplified gnotobiotic gut community in the presence of dietary inulin. Using a multidisciplinary approach, we were able to demonstrate that a phylogenetically distinct *susC*/*susD* homolog gene pair that is present in *Bt-8736* is the genetic determinant of fructan specificity. Our work highlights how structural differences present in dietary polysaccharides such as fructans can result in distinct molecular mechanisms for utilization of these polymers. Levan utilization by B. thetaiotaomicron* VPI-5482* has an absolute requirement for a cell surface endo-levanase, *BT1760*, which degrades the polymer into shorter chains that are channeled through the SusC/D homolog transport apparatus (7). On the other hand, inulin utilization in *Bt-8736* could proceed in the absence of an endo-inulinase, most likely due to the average chain length of inulin being shorter than that of levan, allowing importation without prior depolymerization (14). Such a mechanism would enable utilization of dietary inulin by minimizing its loss to the external environment (15). It was previously reported that the fructan PUL in Bacteroides ovatus encodes cell surface endo-inulinases that enable cross-feeding between the species that were mostly likely to cooperate in the human gut. Interestingly, even in this PUL, the endo-inulinases were dispensable for inulin use by B. ovatus, suggesting a similar mechanism for inulin uptake (16).

Our current work highlights the limitations of functional predications based on 16S rRNA gene relatedness alone. Integration of top-down genomics approaches with bottom-up culture-based microbiology and molecular genetics is proving to be a powerful approach to determine the vast diversity of PULs present in gut *Bacteroidetes* (15–17), many of which are involved in utilization of MACs. Given the significant role played by these MACs in determining gut microbiota structure and dynamics (18), dietary intervention using MACs has a great potential as a noninvasive method for modulating our gut microbes. A key method to enable accurate predictions is that of identifying and characterizing the diverse molecular mechanisms that underlie these MAC utilization pathways.

## MATERIALS AND METHODS

### Bacterial growth analyses.

B. thetaiotaomicron* VPI-5482*, *Bt-8736* (a gift from Eric Martens), *Bt*(*8736-2*), and Bacteroides caccae ATCC 43185 were cultured in tryptone-yeast extract-glucose medium (TYG) and *Bacteroides* minimal medium (MM) containing indicated carbohydrates as described previously (19, 20). MM cultures were inoculated (1:50) from a stationary-phase culture grown in TYG. Growth curves were obtained using a Powerwave spectrophotometer (BioTek) that measured optical density at 600 nm (OD_600_) every 30 min under anaerobic conditions (N_2_, 74%; CO_2_, 20%; H_2_, 6%) at 37°C.

### Carbohydrates used in the study.

Glucose, fructose, sucrose, maltotriose, chicory inulin (average degree of polymerization [dp], ≥25) (for ITC), and levan (L8647) were purchased from Sigma. Orafti HP inulin (dp, ≥23), which was used for the rest of the studies, was obtained from the Beneo-Orafti group.

### Genome sequencing and *de novo* assembly.

*Bt*-8736 was sequenced by whole-genome shotgun sequencing using an Illumina system, which achieved 148-fold coverage. There were 25,128,539 reads that were trimmed to remove reads of poor quality, resulting in 24,896,432 reads with an average length of 35.6 nucleotides. A *de novo* assembly strategy using CLC Genomics Workbench in lieu of a reference assembly with respect to the previously sequenced strain (B. thetaiotaomicron* VPI-5482*) was used to maximize the likelihood of assembling regions that differed between the two strains. This strategy matched 24,660,279 reads into 376 contigs that ranged in length from 200 to 239,349 nucleotides and that had an average length of 15,744 nucleotides and an N50 of 53,056 nucleotides. The total genome size was 5.9 Mb.

### Phylogenetic analysis.

Amino acid sequences from *Bacteroides* SusC and SusD homologs and 16S rRNA were aligned and neighbor-joining phylogenetic trees were generated using MEGA 5.0 software. The accession numbers of all the amino acid sequences used for the analysis are provided in Table S2 in the supplemental material.

### Genetic modification of B. thetaiotaomicron* VPI-5482*.

The fructan PUL* susC*/*susD* homolog gene pair from *Bt-8736*, along with the intergenic 5′ region containing putative promoters (Table S1), was PCR amplified using high-fidelity Platinum Pfx DNA polymerase (Invitrogen). The resulting PCR product was ligated into either a pNBU2-tetQb or a pNBU2-ErmGb vector, transformed by electroporation into Escherichia coli S17.1 λ-pir, and conjugated into B. thetaiotaomicron VPI-5482 (19). The resulting clones were screened by PCR and sequenced to confirm stable site-specific integration in either of the two sites located at the 3′ end of a serine tRNA gene in B. thetaiotaomicron* VPI-5482* (21). In-frame, nonpolar gene deletions for glycoside hydrolase 32 knockouts were generated using a counterselectable allele exchange protocol and specific primers (Table S1) (19).

10.1128/mSphereDirect.00185-18.5 TABLE S1  List of primers used in the study. Download TABLE S1, DOCX file, 0.02 MB. Copyright © 2018 Joglekar et al. 2018 Joglekar et al. This content is distributed under the terms of the Creative Commons Attribution 4.0 International license.

10.1128/mSphereDirect.00185-18.6 TABLE S2  List of accession numbers of amino acid sequences used for the phylogenetic analysis. Download TABLE S2, DOCX file, 0.02 MB. Copyright © 2018 Joglekar et al. 2018 Joglekar et al. This content is distributed under the terms of the Creative Commons Attribution 4.0 International license.

### Cloning and expression of recombinant SusD.

*Bt-8736* fructan PUL SusD, lacking its native signal sequences, was amplified from genomic DNA using the primers listed in Table S1. The construct was cloned into pET28b (Novagen), expressed in E. coli BL21, and purified by immobilized metal affinity chromatography as described previously (22).

### Quantitative RT-PCR.

Gene-specific primers were used to measure expression of the fructan PUL and 16S rRNA genes using the threshold cycle (ΔΔ*CT*) method as described previously (Table S1) (19). *In vivo* expression of the *Bt-8736*s *susC*/*susD* homolog gene pair was quantified by normalizing the expression of each gene to the relative density of *Bt-8736* in these samples. The assay was conducted using Brilliant III Ultra-Fast SYBR green (Agilent) in an MX3000P thermocycler (Stratagene).

### Isothermal titration calorimetry.

Isothermal titration calorimetry (ITC) was carried out at 25°C in 20 mM HEPES buffer (pH 8.0) essentially as described previously (22). Protein concentrations were between 50 and 100 µM in the cell, and glycans (inulin or levan; Sigma) in the syringe were at 1% (wt/vol). Binding curves were fitted to a single-site model in Microcal Origin v7.0 with the “ligand in cell” option selected, as the molar concentration of binding sites on the polymeric glycans was not known.

### TLC and HPAEC analysis.

Anaerobic cultures of *Bacteroides* spp. were grown in MM containing fructose (0.5% [wt/vol]) at 37°C and harvested at mid-log phase (OD_600_ between 0.4 and 0.6). Cells were washed twice with PBS (pH 7.2) to remove residual fructose, resuspended at a 10-fold-lower volume in PBS-containing inulin (0.5% [wt/vol]), incubated aerobically from 0 to 4 h, and placed immediately at −20°C. For thin-layer chromatography (TLC) analysis, samples were spotted onto glass silica plates, air dried, and processed in a glass tank equilibrated with a 1-butanol–2-propanol–acetic acid–H_2_O (7:5:2:4) solvent system (23). Resolved oligosaccharides, disaccharides, and monosaccharides were visualized using orcinol spray reagent (sulfuric acid/ethanol/H_2_O at 3:70:20 [vol/vol], orcinol at 1% [wt/vol]), heating the plates at 100°C for 10 min. Four-hour samples were analyzed by HPAEC at the Glycotechnology Core of the University of California, San Diego, for high-performance anion-exchange chromatography with pulsed amperometric detection (HPAEC-PAD) analysis.

### Reconstituted medium growth curve analysis.

TYG-grown cultures of B. thetaiotaomicron* VPI-5482*, *Bt-8736*, *Bt*(*8736-2*), and B. caccae were inoculated (1:50) into MM-inulin (0.5% [wt/vol]) and grown anaerobically at 37°C. All cultures were harvested at mid-log phase and were filter sterilized to collect the spent medium. B. thetaiotaomicron* VPI-5482* was inoculated as described above into the spent medium, which was reconstituted (1:1) with 2× MM without any carbon source. MM-fructose (2×; 1.0% [wt/vol]) added to the reconstituted medium (1:1) served as a control for the presence of any inhibitory molecules in the spent medium. B. thetaiotaomicron* VPI-5482* was also grown simultaneously in fresh MM-fructose (0% to 0.5% [wt/vol]).

### Enzyme localization studies.

Cultures (5 ml) grown on fructose (0.5% [wt/vol]) were harvested by centrifugation (OD_600_ of ~1.0). Cells were washed with PBS and then resuspended in 1 ml 20 mM Tris-HCl (pH 8.0) for whole-cell assays. Washed cells were also used to make the periplasmic fraction, which was prepared as follows. Washed cells from 5-ml cultures were resuspended in 5 ml of ice-cold 20% sucrose–20 mM Tris-HCl (pH 7.5)–1 mM EDTA and incubated on ice for 30 min before being pelleted by centrifugation. Cells were then resuspended in 1 ml ice-cold 1 mM MgCl_2_–PBS and centrifuged, and the supernatant was retained as the periplasmic fraction. This periplasmic fraction was then extensively dialyzed in PBS to remove contaminating sucrose. For enzyme assays, 100 µl of washed whole cells or the periplasmic fraction was added to cuvettes containing PBS-washed cells and 0.5% inulin (final concentration)–20 mM Tris-HCl (pH 8.0) at 37°C and fructose release was monitored continuously using a modified fructose detection kit (Megazyme International). Activities of the periplasmic marker alkaline phosphatase and cytoplasmic marker glucose-6-phosphate dehydrogenase were compared to the results seen with lysed cells to ensure that no cell lysis/leakage had occurred.

### Gnotobiotic mouse experiments.

Germfree Swiss-Webster mice were maintained in gnotobiotic isolators and fed an autoclaved standard diet (Purina LabDiet 5K67) or a custom diet (Bio-Serv) (7), in accordance with the Administrative Panel on Laboratory Animal Care (A-PLAC), the Stanford University School of Medicine IACUC. Mice were bicolonized using a single oral gavage dose of 10^8^ to 10^9^ CFU of each strain from stationary-phase cultures grown in TYG. Fecal samples were collected on days 4, 7, 14, and 21, and total fecal DNA was extracted using the phenol-chloroform-isoamyl alcohol method, followed by a cleanup performed using a Qiagen DNeasy blood and tissue kit. Bacterial densities were determined by real-time quantitative PCR using strain-specific primers (Table S1).

### Data availability.

This Whole Genome Shotgun project has been deposited at DDBJ/ENA/GenBank under accession number QAJH00000000. The version described in this paper is version QAJH01000000. Note that NCBI genome classification has assigned *Bt-8736* as a Bacteroides faecis based on whole-genome similarity. The 16S rRNA gene sequence of *Bt-8736* and B. faecis are both >97% identical to the B. thetaiotaomicron type strain (24).
