# Supplementary figures and images for: Genetic Variation of the SusC/SusD Homologs from a Polysaccharide Utilization Locus Underlies Divergent Fructan Specificities and Functional Adaptation in Bacteroides thetaiotaomicron Strains
Source: mSphere. 2018 May 23;3(3):e00185-18. doi: 10.1128/mSphereDirect.00185-18 (PMC5967196; doi:10.1128/mSphereDirect.00185-18)

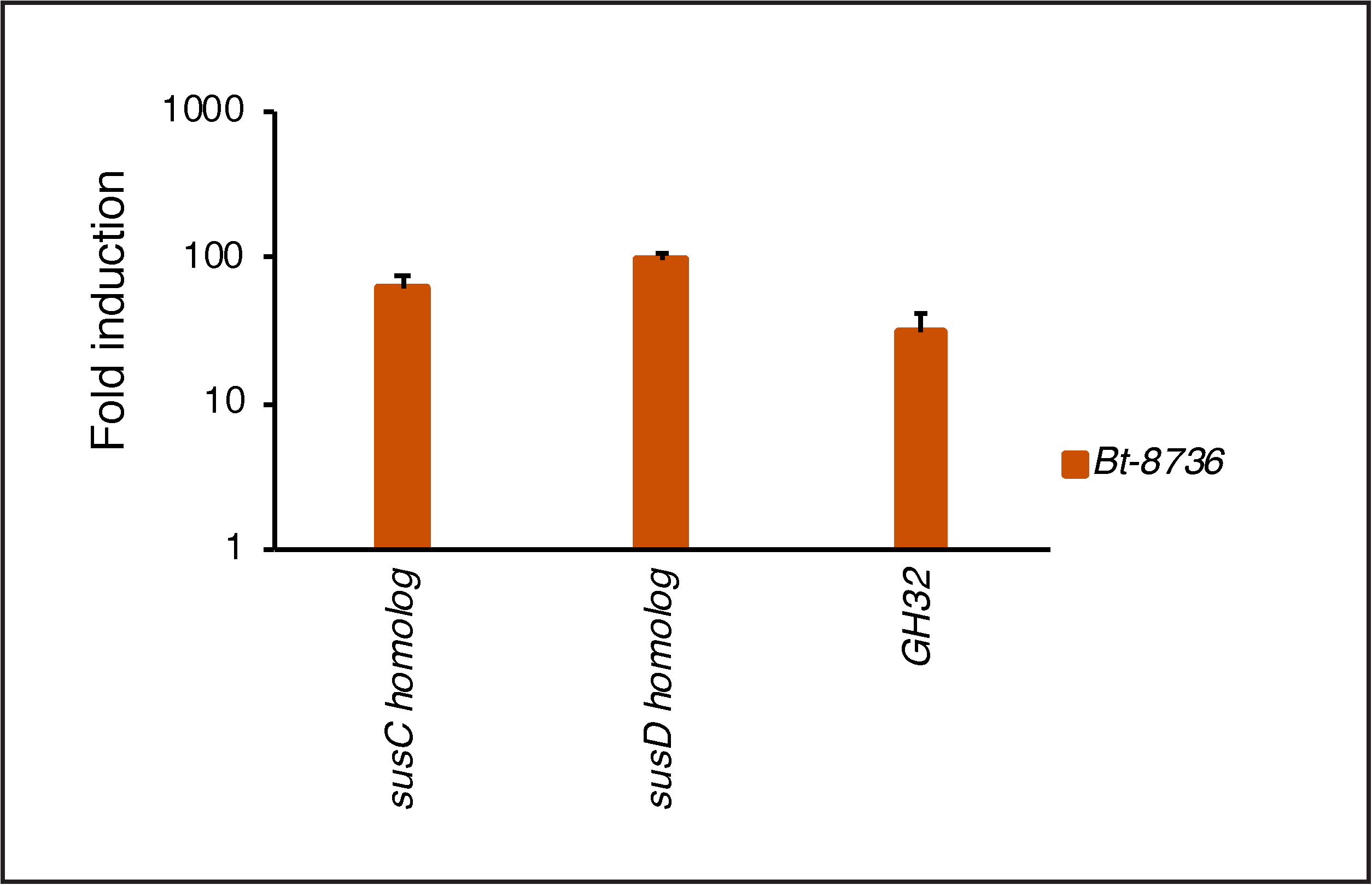

Supplement: FIG S1 [file sph003182546sf1.tif]

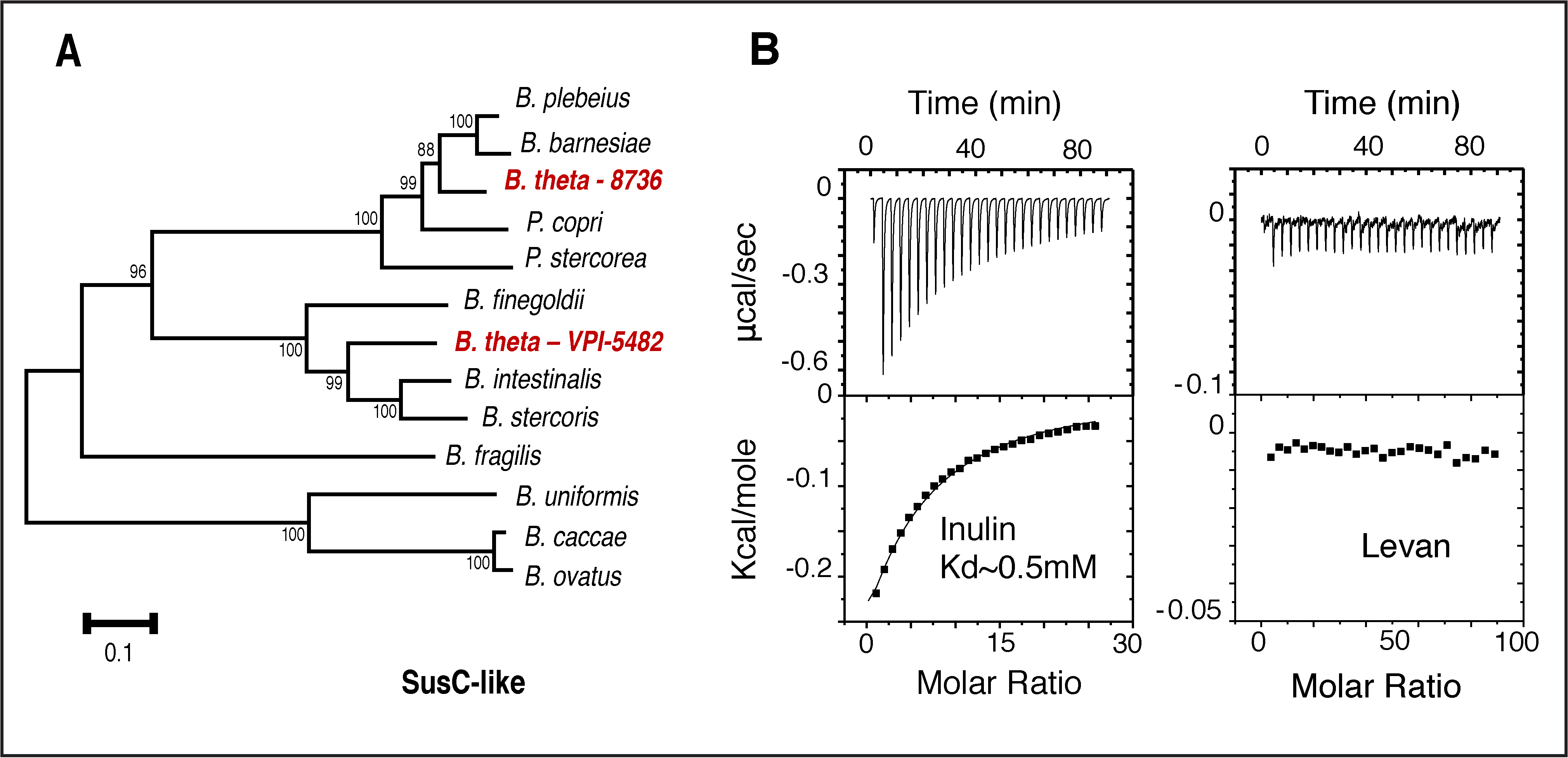

Supplement: FIG S2 [file sph003182546sf2.tif]

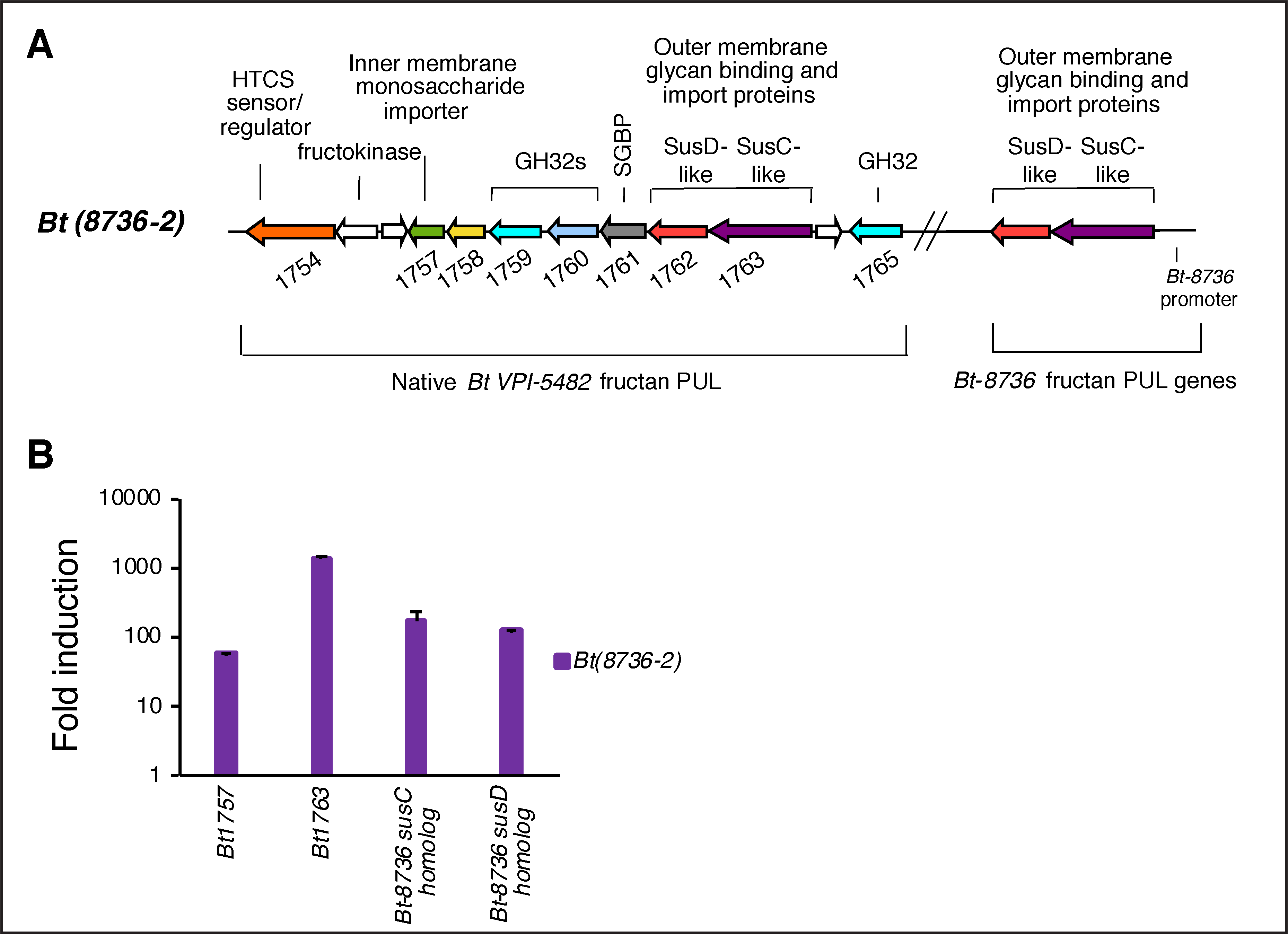

Supplement: FIG S3 [file sph003182546sf3.tif]

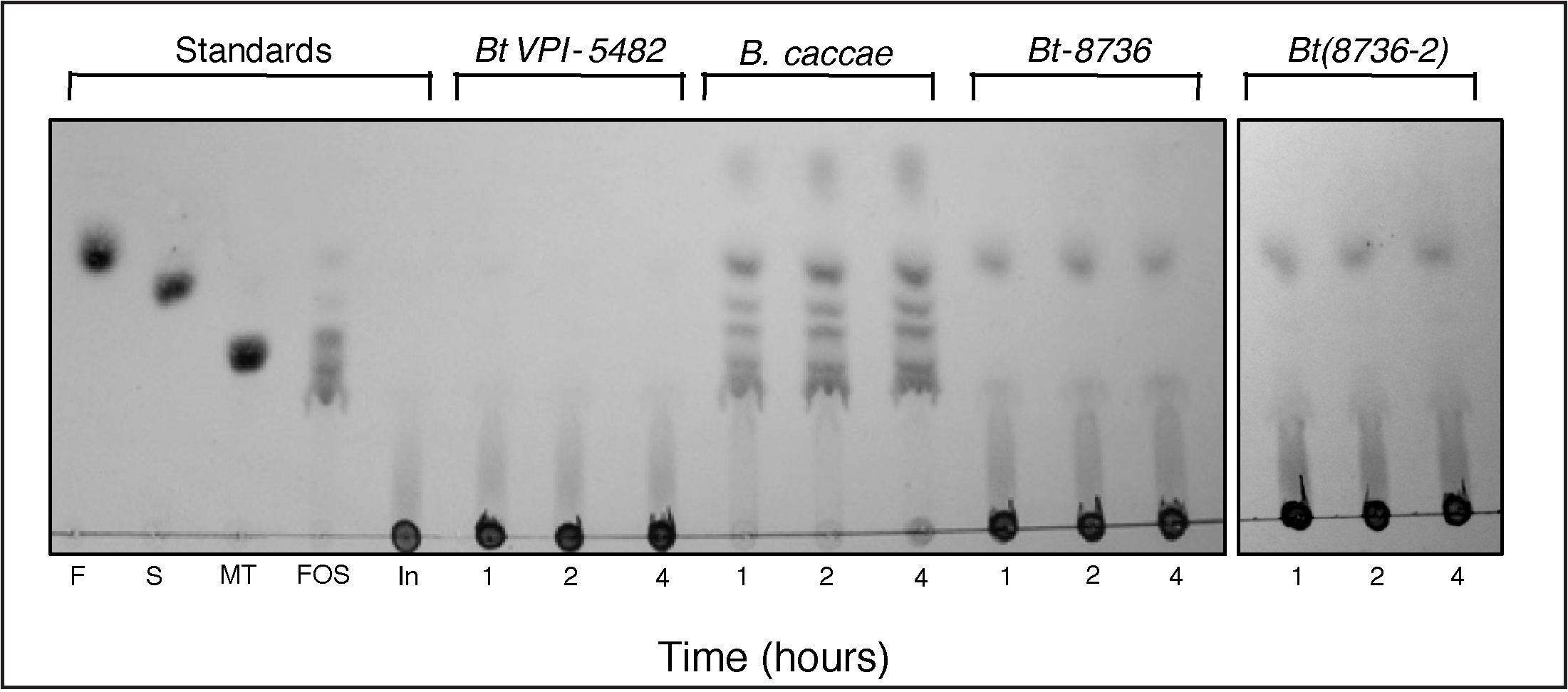

Supplement: FIG S4 [file sph003182546sf4.tif]
