# Supplementary material for: Genetic Variation of the SusC/SusD Homologs from a Polysaccharide Utilization Locus Underlies Divergent Fructan Specificities and Functional Adaptation in Bacteroides thetaiotaomicron Strains
Source: mSphere. 2018 May 23;3(3):e00185-18. doi: 10.1128/mSphereDirect.00185-18 (PMC5967196; doi:10.1128/mSphereDirect.00185-18)
Supplement: TABLE S1 [file sph003182546st1.docx]

**Table S1. List of primers used in the study**

|  | ***Bt* mutants** | **Primers** |
| --- | --- | --- |
| 1. | ***BtΔ1759*** |  |
|  | BtΔ1759_1000up | AAAAGGATCCCATGATGTGGGATCGTTTCTAC |
|  | BtΔ1759_750up | AAAAGGATCCGGTGTCCCACACGTGCC |
|  | BtΔ1759_700up | AAAAGGATCCAGAACCCGAATGGGCAGG |
|  | BtΔ1759_sewing_forward | TAACAACCTTAATACCATAGAACATGTAAAAAACGACTTTCTTCTCCCTG |
|  | BtΔ1759_sewing_reverse | CAGGGAGAAGAAAGTCGTTTTTTACATGTTCTATGGTATTAAGGTTGTTA |
|  | BtΔ1759_1000down | AAAATCTAGATCAGAATCTTAGGTGCAGTAGT |
|  | BtΔ1759_750down | AAAATCTAGACCCCACATGGCAATGTACG |
|  | BtΔ1759_700down | AAAATCTAGA GTCCGAAGGTGAGGCTGC |
| 2. | ***BtΔ1765*** |  |
|  | BtΔ1765_1000up | AAAAGGATCCTCTTGTCCAATGTCGATGCTT T |
|  | BtΔ1765_750up | AAAAGGATCCGGATATCACTCGTGACGATG |
|  | BtΔ1765_700up | AAAAGGATCCTATTTGATATTTTGTCTGCAATCG A |
|  | BtΔ1765_sewing_forward | CTATTGAAAAATAAATCCATCCTTTTACATGGTAACAGGATATTCAGAGT |
|  | BtΔ1765_sewing_reverse | ACTCTGAATATCCTGTTACCATGTAAAAGGAT GATTTATTTTTCAATAG |
|  | BtΔ1765_1000down | AAAATCTAGATGG GAAAGAGTATCTGTGGCA |
|  | BtΔ1765_750down | AAAATCTAGAATAGGTTATCGTATTCAGGGT AA |
|  | BtΔ1765_700down | AAAATCTAGAGAATACCGGAGAAAAAGAAATGTA |
| 3. | ***BtΔ3082*** |  |
|  | BtΔ3082_1000up | AAAAGGATCCTCAATATATGTGCTCTGTCAAAGA |
|  | BtΔ3082_750up | AAAAGGATCCCCCTCTCAATTGGCGAAAGA |
|  | BtΔ3082_700up | AAAAGGATCCTCCCGGAGCACGTGCGC |
|  | BtΔ3082_sewing_forward | AAAGATAGCTTTTGATTTCCGTCTACATAGCTAT TTTATTTATTAGTTTGTAAAA |
|  | BtΔ3082_sewing_reverse | TTTTACAAACTAATAAATAAAATAGCTATGTAG ACGGAAATCAAAAGCTATCTT T |
|  | BtΔ3082_1000down | AAAATCTAGATAG ATGCATAGCGGGATTAG |
|  | BtΔ3082_750down | AAAATCTAGAGGTAGGCTCGATGATCGTTG |
|  | BtΔ3082_700down | AAAATCTAGAGGCATCTTCAATCATTGCCAAT |
| 4. | ***Bt(8736-2)*** |  |
|  | Bt(8736-2)_forward | AAAAGCGGCCGCTCACAAACCGTAACAGGTCGTCAC |
|  | Bt(8736-2)_reverse | AAAAGGATCCTCAATAATTCTTATTTTGAGTATATAATCCGGGAAT |
|  | **Quantitative RT-PCR** |  |
| 1. | Bt16S_forward | GTGTAGCGGTGAAATGCTTAGATATC |
|  | Bt16S_reverse | CAGTGTCAGTTGCAGTCCAGTGA |
| 2. | Bt1757_forward | GTCTTTCAAACGCTGCAACA |
|  | Bt1757_reverse | GAGCTTCGGGAACAGACTTG |
| 3. | Bt1763_forward | ATGCCTGGTCACCTACGAAC |
|  | Bt1763_reverse | CAAGCGGTCCATTCTCATTT |
| 4. | Bt-8736-susClike_forward | CAGCAACAGGAACAACGAGA |
|  | Bt-8736-susClike_reverse | ACGATTGTCCTTGCCAAAAC |
| 5. | Bt-8736-susDlike_forward | ACTGGGTGAACTGGGTGAAG |
|  | Bt-8736-susDlike_reverse | CGCAGACATTTACGTGCTGT |
| 6. | Bt-8736-GH32_forward | TGCTGGAAAGAAATCAGGCT |
|  | Bt-8736-GH32_reverse | TGCATCAGGTCTTTGCTGAC |
|  | **Quantitative PCR** |  |
| 1. | Bt_forward | ATGCCTGGTCACCTACGAAC |
|  | Bt_reverse | CAAGCGGTCCATTCTCATTT |
| 2. | Bt-8736_forward | CAGCAACAGGAACAACGAGA |
|  | Bt-8736_reverse | ACGATTGTCCTTGCCAAAAC |
|  | **Cloning and expression in *E. coli*** |  |
| 1. | Bt8736_SusD-forward | CTCGCTAGCGATGATTTTCTAGACTATAGTCCAAC |
|  | Bt8736_SusD-reverse | CTCCTCGAGATAATTCTTATTTTGAGTATATAATCCG |
