# Supplementary material for: Genetic Variation of the SusC/SusD Homologs from a Polysaccharide Utilization Locus Underlies Divergent Fructan Specificities and Functional Adaptation in Bacteroides thetaiotaomicron Strains
Source: mSphere. 2018 May 23;3(3):e00185-18. doi: 10.1128/mSphereDirect.00185-18 (PMC5967196; doi:10.1128/mSphereDirect.00185-18)
Supplement: TABLE S2 [file sph003182546st2.docx]

**Table S2. List of Accession numbers of amino acid sequences used for the phylogenetic analysis**

| **Species name** | **Accession number** | | | |
| --- | --- | --- | --- | --- |
|  | **16S rRNA** | **SusD-like** | | **SusC-like** |
| *B. plebeius* | AB200217.1 | EDY93611.1 | | WP_007564554.1 |
| *B barnesiae* | AB253726.1 | WP_034776085.1 | | CDD32774.1 |
| *P. stercorea* | AB244774.1 | WP_007901225.1 | | CDE32916.1 |
| *P. copri* | AB064923.2 | EFB34080.1 | | WP_006849158.1 |
| *B. finegoldii* | AB222699.1 | WP_007748321.1 | | EDS13293.1 |
| *B. theta VPI* 5482 | NR_074277.1 | AAO76869.1 | | AAO76870.1 |
| *B. theta* 8736^1^ | QAJH00000000 |  | |  |
| *B. intestinalis* | NR_041307.1 | EDV04070.1 | | EDV04069.1 |
| *B. stercoris* | NR_027196.1 | EDS13294.1 | | EDS13293.1 |
| *B. uniformis* | NR_040866.1 | EDO55066.1 | | EDO55065.1 |
| *B. caccae* | NR_026242.1 | EDM19973.1 | | EDM19972.1 |
| *B. ovatus* | NR_119165.1 | | EDO10123.1 | EDO10124.1 |
| *B. fragilis* | KP326374.1 | | CAH09989.1 | CAH09990.1 |

^1^ This Whole Genome Shotgun project has been deposited at DDBJ/ENA/GenBank under the accession QAJH00000000. The version described in this paper is version QAJH01000000.
